# Combination Effects of (Tri)Azole Fungicides on Hormone Production and Xenobiotic Metabolism in a Human Placental Cell Line

**DOI:** 10.3390/ijerph110909660

**Published:** 2014-09-17

**Authors:** Svenja Rieke, Sophie Koehn, Karen Hirsch-Ernst, Rudolf Pfeil, Carsten Kneuer, Philip Marx-Stoelting

**Affiliations:** Federal Institute for Risk Assessment (BfR), Max-Dohrn-Str 8-10, 10589 Berlin, Germany; E-Mails: svenja.rieke@bfr.bund.de (S.R.); sophie.koehn@bfr.bund.de (S.K.); Karen-ildico.hirsch-ernst@bfr.bund.de (K.H.-E.); rudolf.pfeil@bfr.bund.de (R.P.); carsten.kneuer@bfr.bund.de (C.K.)

**Keywords:** mixture toxicity, endocrine disruption, placenta, triazoles

## Abstract

Consumers are exposed to multiple residues of different pesticides via the diet. Therefore, EU legislation for pesticides requires the evaluation of single active substances as well as the consideration of combination effects. Hence the analysis of combined effects of substances in a broad dose range represents a key challenge to current experimental and regulatory toxicology. Here we report evidence for additive effects for (tri)azole fungicides, a widely used group of antifungal agents, in the human placental cell line Jeg-3. In addition to the triazoles cyproconazole, epoxiconazole, flusilazole and tebuconazole and the azole fungicide prochloraz also pesticides from other chemical classes assumed to act via different modes of action (*i.e.*, the organophosphate chlorpyrifos and the triazinylsulfonylurea herbicide triflusulfuron-methyl) were investigated. Endpoints analysed include synthesis of steroid hormone production (progesterone and estradiol) and gene expression of steroidogenic and non-steroidogenic cytochrome-P-450 (CYP) enzymes. For the triazoles and prochloraz, a dose dependent inhibition of progesterone production was observed and additive effects could be confirmed for several combinations of these substances *in vitro*. The non-triazoles chlorpyrifos and triflusulfuron-methyl did not affect this endpoint and, in line with this finding, no additivity was observed when these substances were applied in mixtures with prochloraz. While prochloraz slightly increased aromatase expression and estradiol production and triflusulfuron-methyl decreased estradiol production, none of the other substances had effects on the expression levels of steroidogenic CYP-enzymes in Jeg-3 cells. For some triazoles, prochloraz and chlorpyrifos a significant induction of *CYP1A1* mRNA expression and potential combination effects for this endpoint were observed. Inhibition of *CYP1A1* mRNA induction by the AhR inhibitor CH223191 indicated AhR receptor dependence of this effect.

## 1. Introduction 

Consumers are exposed to multiple residues of different pesticides via the diet. In fact, in 2010 about a quarter of agriculture products tested in the EU contained more than one pesticide residue [1]. Since the current authorisation of pesticides is performed for individual active substances, the occurrence of multiple residues raises questions concerning potential combination effects, especially for substances causing toxicity by a common mode of action [2]. Pesticides acting by a common mode of action have previously been shown to act in a cumulative manner, often with different potencies [3,4,5,6,7,8]. One group of pesticides acting by such a common mode of action, for which additive effects have been postulated, are the triazole fungicides.

Triazoles belong to the most used agricultural fungicides due to their efficacy as well as their curative and eradicative properties [9]. The fungicidal activity of azole fungicides is due to an inhibition of the fungal cytochrome P-450-(CYP)-enzyme ergosterol synthetase. Since most triazoles are not completely specific, they also inhibit mammalian CYP-enzymes [10,11]. Especially aromatase, an enzyme that converts androgens to estrogens, e.g. testosterone to estradiol, is a known target for unspecific inhibition by triazoles, but other CYP-enzymes, many of which contribute to steroid biosynthesis, are also affected. Several experiments have shown that triazoles are able to act in a dose additive manner regarding CYP-enzyme inhibition [6,12,13].

While most triazoles exhibit profound hepatotoxic abilities, several are also classified for their reproductive and/or developmental toxicity [14]. To cause direct embryotoxic effects azole fungicides have to reach the foetus via the placenta. Placental metabolism and hormone production may also contribute to the overall developmental toxicity of azole fungicides. In fact, it has been postulated that placental hormone production and metabolism may be a key event in the mode of action of epoxiconazole and may also explain species differences in reproductive toxicity between rats and humans [15]. Pregnant rats fed with different triazoles exhibit significantly increased postimplantation and perinatal loss coupled with increased progesterone plasma hormone levels at GD 21 [16]. Increased progesterone levels often cause prolonged gestation times, which were also reported in several draft assessment reports for triazole fungicides, e.g., by EFSA [17]. With regard to combination effects, mixtures containing triazoles applied below the individual NOAELs caused impaired parturition and other developmental effects in rats that were not observed when the substances were administered individually at the same dose level [5]. During pregnancy, the steroid hormone progesterone is mainly produced by the placental trophoblast. Hence, it seems reasonable to use a placental cell line as a model for cumulative effects of these substances on hormone production. An appropriate model where synthesis of steroid hormones occurs is the human choriocarcinoma cell line Jeg-3, which resembles cells of the human trophoblast at the first trimester [18].

Within this study, combination effects of (tri)azole fungicides on the production of progesterone and estradiol were analysed using the Jeg-3 model. To test whether pesticides, belonging to different chemical classes and exhibiting different pesticidal modes of action, might contribute to common effects as suggested e.g., by Kortenkamp *et al.* [19], combinations of the azole prochloraz, chlorpyrifos and triflusulfuron-methyl were also examined.

Besides the ability to inhibit CYP-enzyme and the resulting impact on steroid synthesis, triazole fungicides are also able to interact with nuclear receptors, especially with the androgen and estrogen receptors with different specificities [11,20,21,22]. Again, several triazoles were demonstrated to exhibit dose additive effects when applied in a mixture [23,24,25,26]. In addition to the well examined androgene and estrogene receptors, the placenta also expresses aryl-hydrocarbone receptor (AhR) [27]. While this receptor is known to be a major player in xenobiotic response in the liver, recent studies indicate an important role for AhR in pregnancy, fetal growth and neonatal survival. For example, the ability of female AhR knockout mice to establish implantation and maintain pregnancy was compromised [28,29,30,31]. They exhibit decreased litter size and increased neonatal death. On the other hand, increased AhR expression upon xenobiotic stimulation leads to morphological changes in the placenta vascularisation, which is not apparent in AhR deficit mice [32,33]. For some substances, an effect on the dilatation of the maternal blood sinusoids and the maternal blood flow has been shown. Even though triazoles are known activators of the constitutive androstane receptor (CAR) in liver some triazoles used as pharmaceuticals have been shown to interact directly with the AhR in different hepatic cell lines, thus changing the expression of AhR dependent genes [34]. Since a large number of substances of different chemical classes is able to interact with AhR, possible combination effects on AhR activation in the placenta might occur. Therefore, we additionally examined combination effects of triazoles, the azole fungicide prochloraz, the herbicide triflusulfuron and the insecticide chlorpyrifos regarding AhR activation. Chlorpyrifos is an organophosphate insecticide that has previously been shown to be an AhR agonist [35]. Additionally, chlorpyrifos has been reported to cause effects on ovarian and mammary gland tissues in a non-guideline rat developmental toxicity study [36] and more generally developmental and reproductive effects in mice and rats [37,38]. In contrast triflusulfuron is an herbicide, which was found not to be toxic for reproduction but to cause Leydig cell tumors in rats [39]. Mechanistic studies provided evidence on interference of the active substance with CYP19 (aromatase) activity [39]. Hence the pesticidal modes of action of both substances differ from that of triazoles.

The obvious multitude of possible chemical combinations prevents the analysis of pesticide mixtures in routine regulatory toxicity testing, as they are carried out for individual active substances. An applicable method for the investigation of mixture effects may be the development of *in vitro* models for target organ toxicity, since this would allow high throughput testing of many combinations and might also reveal mechanistic information. At present, efforts regarding the implementation of mixture toxicity in regulatory authorisation of pesticides are focussing on target organ-based cumulative assessment groups [40]. Clustering of pesticides according to the specific mode of action is also considered for refinement but frequently not feasible because of limited information on the mode of action of many active substances in general and because of uncertainty on whether or not different modes of action contributing to a common outcome are independent from one another.

To analyse potential combination effects of substances with similar and dissimilar mode of action on two distinct endpoints (progesterone production and *CYP1A1* expression) and to contribute to the development of *in vitro* methods for the analysis of mixture effects we analysed several pesticides in Jeg-3 cells individually and in combination. Here we report the results of this study.

## 2. Experimental Section 

Test substances and inhibitors: flusilazole, tebuconazole, chlorpyrifos and triflusulfuron and the AhR inhibitor CH223191 were purchased from Sigma Aldrich, Traufkirchen, Germany at analytical grade. Cyproconazole (Syngenta, Münchwilen, Switzerland), epoxiconazole, and prochloraz (both BASF, Limburger Hof, Germany) were purchased from the respective manufacturer at technical grade. The following substances/lots were used: azole fungicides: cyproconazol (batch CHF1E00042), epoxiconazole (Lot 5154X), flusilazole (Lot SZB8294XV), prochloraz (batch COD-000718), tebuconazol (Lot SZBB055XV). Other substances: chlorpyrifos (Lot ZBD157XV), triflusulfuron (Lot SZBC327XV). AhR inhibitor CH223191 (Lot 092M4627V).

Cell culture and treatment: Jeg-3 cells were obtained from ATCC (HTB-36, Manassas, VA, USA). They were sub-cultivated on 75 cm^2^ cell culture flasks (tpp, Trasadingen, Switzerland) in MEM medium with phenol red (PAN, Aidenbach, Germany) containing 10% FCS (PAN) at 37 °C/5% CO_2_ in a Binder cell culture incubator. For substance treatment, cells were seeded at 10^5^ cells per well in 6-well plates (tpp, Trasadingen, Switzerland) for subsequent reporter-gene-analysis, hormone measurement and RNA isolation or at 5 × 10^3^ cells per well in 96 well plates (Nunc, Thermo Fisher Scientific, Waltham, MA, USA) for cytotoxicity analysis, and incubated for 24 h prior to treatment in MEM without phenol red and with 5% of charcoal striped (hormone free) FCS (PAN, Aidenbach, Germany). Thereafter, cells were treated with a broad concentration range (0.01 µM to 40 µM) of the respective substances individually or in combination for another 48 h, with 1% DMSO as solvent in the final dilution, due to the poor solubility of some of the substances. The inhibitor CH223191 was applied in a concentration of 10 µM. Control wells contained the same concentration of DMSO (1%) as the treated wells.

Cytotoxicity Assay: cytotoxicity was analysed by WST1-Assay (Roche, Berlin, Germany) according to the standard protocol provided by the supplier. In brief, 10 µL of WST1 reagent was added per well to cells treated with the substances and incubated for 1 h at 37 °C. After incubation, the formation of the formazan was measured on an Infinite M200pro plate reader (Tecan, Maennedorf, Swiss) at 450 nm and compared to untreated as well as positive controls. The cytotoxicity assay was performed for all substances/inhibitors individually and in combination. To ensure specificity of effects, other endpoints were measured in the sub-cytotoxic range.

Hormone measurements: Hormone measurement was performed by ELISA. Progesterone concentration was measured from cell culture supernatant using Progesterone ELISA EIA Kit (Cayman Chemical Ann Arbor, MI, USA). Estradiol concentration was measured from cell culture supernatant using an Estradiol ELISA EIA Kit (Cayman Chemical-/ Demeditec, Kiel, Germany). Absorption was measured on an Infinite M200pro plate reader (Tecan) at 412 nm for the progesterone ELISA EIA and 450 nm for the estradiol ELISA EIA according to the respective manufacturer’s protocol.

Gene expression analysis: RNA was isolated from cells using TRIzol® reagent (Invitrogen, Carlsbad, CA, USA) according to the manufacturers’ protocol. Quality and quantity of RNA samples were controlled with a Nanodrop spectrophotometer (peqLab, Erlangen, Germany). If necessary, RNA was further purified using a RNA purification kit (Qiagen, Venlo, Netherlands). Reverse transcription of 1 µg RNA was then performed using High Capacity cDNA Reverse Transcription Kit and protocol (Applied Biosystems, Thermo Fisher Scientific). For gene expression analysis, quantitative real-time PCR was performed on an ABI 7900HT instrument using SYBR green mastermix (Qiagen) and primers (0.25 µM, sequences shown in Table 1). Relative expression ratios were calculated according to Pfaffl equation by use of *GAPDH* and *18sRNA* as housekeeping genes.

**Table 1:** Sequences of primers used for RT-PCR analysis. Amplification was performed as follows: 10 min 95 °C, 40 cycles: 10 s 95 °C, 15 s 60 °C, 20 s 72 °C Melting curves were recorded to control for identity and specificity.

	Forward Primer	Reverse Primer
*18sRNA*	CGGCTACCACATCCACGGAA	GCTGGAATTACCGCGGCT
*GAPDH*	CCACTCCTCCACCTTTGAC	ACCCTGTTGCTGTAGCCA
*CYP11A1*	GAGACATGGCCACGATGCTA	CCACTTGCACCAGTGTCTTG
*CYP1A1*	TTTGAGAAGGGCCACATCCG	AGGCCTCCATATAGGGCAGAT
*CYP17A*	CTATGCTCATCCCCCACAAGG	GGATTCAAGAAACGCTCAGGC
*CYP19*	TCCCTGTGGACTCTAAATTGCC	TGGGAGATGAGGGGTCCAAT
*CYP1A2*	CGGTGATTGGCAGAGATCGG	GTCCCTCGTTGTGCTGTGG
*ABCC1*	GATCCGCTCTGGGATTGGAA	GTAGAAGAGGTCTGCCCAGC
*UGT2B4*	TTCGGGTTGCAGCCCACGAC	TGGGTTTCCCAGCTTCCAGCCT
*UGT2B17*	AAGCCAAGGGAGCAGCCCTCA	CGCAGGCCAGCAGGAATGCT

Reporter gene assay: Reporter gene activity was analysed by DualLuciferase Assay (Promega, Madison, WI, USA) according to the suppliers protocol. In brief, an AhR reporter gene construct (Qiagen, Venlo, Netherlands) containing an AhR responsive element and the firefly luciferase gene as a reporter were transiently transfected into Jeg-3 cells using TransIT-2020 transfection reagent (Mirus Bio LLC, Madison, WI, USA) alongside with a renilla-luciferase construct as an internal standard. Luciferase activity was measured on a plate reader (Berthold, Bad Wildbach, Germany) according to the Dual Luciferase Assay protocol provided by the supplier.

Statistics: Statistical analysis was performed using parametric standard tests by SigmaPlot for Windows software (Version 11.0, Systat Software Inc., Point Richmond, CA, USA, 2008) and Microsoft Excel 2007. Students t-test was applied to compare two groups. To obtain information on additivity of effects a curve was modelled based on the data obtained for the individual substances assuming additivity. The measured combination effect was than compared to the model, and a model deviation ratio (MDR) of two was accepted as a maximum deviation to accept additivity as suggested by Belden *et al.* [41] as opposed to synergism or antagonism.

## 3. Results 

### 3.1. Effects of Triazole Fungicides Individually and in Combination on Progesterone Production by Jeg-3 Cells

**Figure 1:**
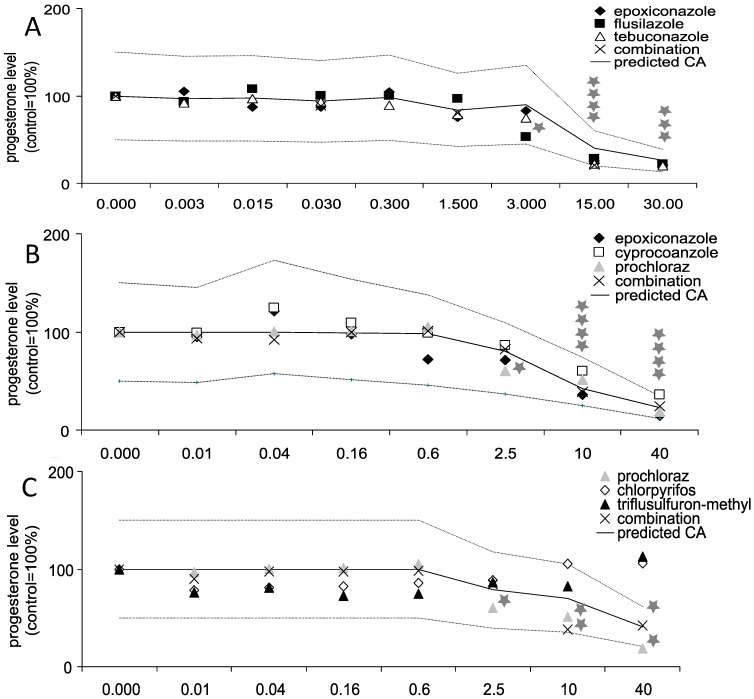
Influence of active substances alone or in combination on progesterone production in Jeg-3 cells. Jeg-3 cells were grown in 6-well cell culture plates for 24 h. Thereafter, the cells were incubated with the substances in concentrations as indicated for 48 h individually and in combination: (**A**) flusilazole, epoxiconazole and tebuconazole, (**B**) epoxiconazole, cyproconazole and prochloraz, (**C**) prochloraz, chlorpyrifos and triflusulfuron-methyl. Finally, the supernatant was collected and progesterone concentration measured by ELISA as indicated by the manufacturers’ protocol. The data represent the mean ± SD for three independent experiments. Concentration values given for combination treatments reflect total concentrations of all three components. Predicted additivity (CA) indicates the calculated combination effect when additivity is assumed. The upper and the lower lines around the predicted CA indicate the MDR (model deviation ratio) based on the predicted CA. Concentrations are given in µM. For graphical clarity error bars were abandoned. The mean standard deviation was about 15%, with the exemption of one combination group (B), where the mean standard deviation turned out to be 30%. Concentration values given for combination treatments reflect total concentrations for all three components. * Indicates significance with p ≤ 0.05.

The main steroid produced in the human placenta is progesterone. While in the first trimester of pregnancy, the ovary is contributing to progesterone synthesis, the human placenta is the major producer in the last two trimesters. In our study using the human placental cell line Jeg-3 all triazoles as well as the azole fungicide prochloraz showed a significant decrease in progesterone secretion starting at 2.5 µM for prochloraz (60% of control; *p* ≤ 0.01), 10 µM for epoxiconazole (36%; *p* ≤ 0.01) and cyproconazole (60%; *p* ≤ 0.01), 3 µM for flusilazole (53%; *p* ≤ 0.05) and 15 µM for tebuconazole (22%; *p* ≤ 0.01, Figure 1). No changes in progesterone secretion were shown for chlorpyrifos and triflusulfuron-methyl. In all combination experiments, in which the concentrations were one-third of the concentrations mentioned above, mixture effects on progesterone secretion were noted. The results of the experiments were compared to a predicted dose response curve based on the individual effects and assuming additivity as a model. The mixture effect of the combinations containing epoxiconazole, flusilazole and tebuconazole was practically identical to effects predicted by the calculated dose response curve (20% *vs.* 22% at 15 µM; *p* ≤ 0.05 as compared to solvent control). The same was observed for the mixture containing epoxiconazole, cyproconazole and prochloraz (24% *vs.* 23% at 40 µM; *p* ≤ 0.05 as compared to solvent control) and the mixture effect for prochloraz, chlorpyrifos and triflusulfuron-methyl (42% *vs*. 41% at 40 µM; *p* ≤ 0.05 as compared to solvent control) No decreased cell viability, measured via the WST1 assay, was observed in the tested concentration range for any substance, indicating specificity of the observed effects.

### 3.2. Effects of Triazole Fungicides Individually and in Combination on Estradiol Production by Jeg-3 Cells

**Figure 2:**
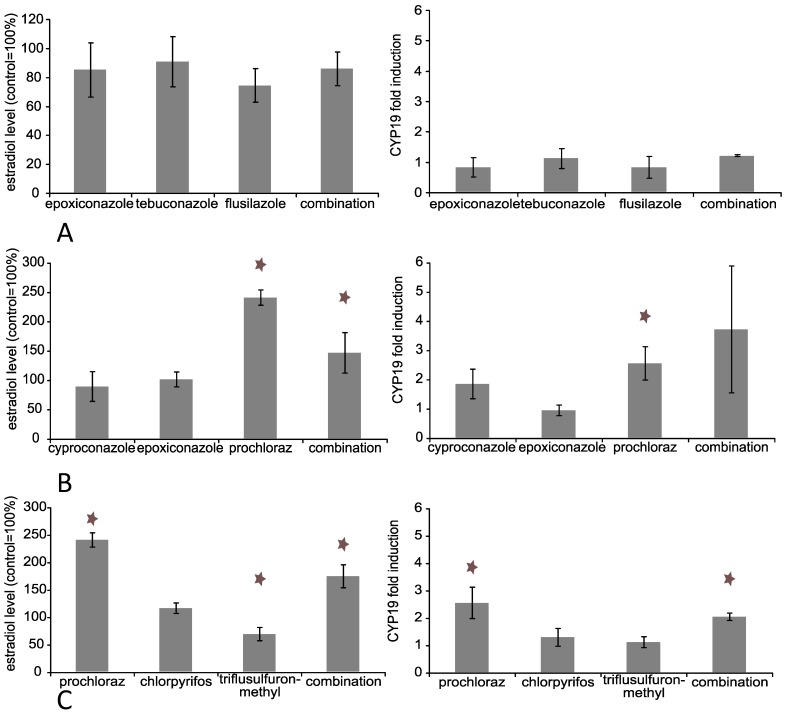
Influence of active substances alone or in combination on estradiol production and *CYP19* mRNA expression in Jeg-3 cells. Jeg-3 cells were grown in 6-well cell culture plates for 24 h. Thereafter, the cells were incubated with the substances in total concentrations of 30 and 40 µM as indicated for 48 h individually and in combination: (**A**) 30 µM for epoxiconazole, tebuconazole and flusilazole, (**B**) 40 µM for epoxiconazole, cyproconazole and prochloraz, (**C**) 40 µM for prochloraz, chlorpyrifos and triflusulfuron-methyl. The supernatant was collected and estradiol concentration measured by ELISA as indicated by the manufacturer’s protocol. The RNA of the cells was isolated and reverse transcription performed as described. The data represent the mean for three independent experiments. * Indicates significance with p ≤ 0.05.

No change in the estradiol concentration was detectable after treatment with any individual triazole used in this experiment (Figure 2). Surprisingly, incubation with 40 µM of the azole fungicide prochloraz led to a significant increase of estradiol to about 250% (*p* ≤ 0.01) of control. 40 µM triflusulfuron-methyl decreased the estradiol concentration slightly but significantly (70% of control; *p* ≤ 0.01). The combination without prochloraz exhibited no changes in the estradiol concentration at 40 µM, while the combinations containing prochloraz showed increased levels: 150% of control (*p* ≤ 0.01) for cyproconazole, epoxiconazole and prochloraz and 175% (*p* ≤ 0.01) for prochloraz, chlorpyrifos and triflusulfuron-methyl. Overall, the amount of estradiol in the supernatants of Jeg-3 cells was quite low (control: 6 pg/mL), but still detectable with a sensitive ELISA.

### 3.3. Effects of Triazole Fungicides Individually and in Combination on Steroid Biosynthesis Dependent Gene Expression

**Figure 3:**
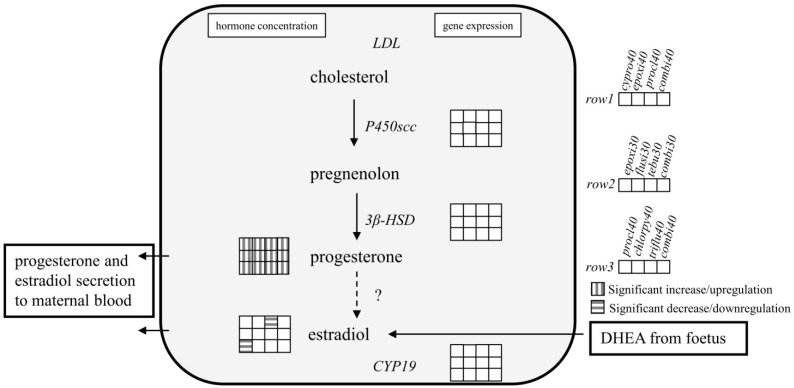
Scheme of expression of steroid biosynthesis relevant genes and supernatant hormone concentration of progesterone and estradiol in trophoblasts based on experiments in Jeg-3 cells. Jeg-3 cells were treated with either 30 µM or 40 µM (as indicated) of cyproconazole, chlorpyrifos, epoxiconazole, flusilazole, prochloraz, tebuconazole and triflusulfuron-methyl individually and in combination for 48 hours. Gene expression was analysed by quantitative real-time PCR. Progesterone and estradiol were measured using commercial ELISA systems. Key on the right side indicates the order of presentation for the concentration groups as well as significant increase/upregulation and decrease/downregulation. The data represent the mean ± SD for three independent experiments. Concentration values given for combination treatments reflect total concentrations of all three components.

In contrast to peptide hormones, steroid hormones are not stored in cellular bodies and released upon a specific stimulus, but have to be synthesised immediately upon requirement. Thus, steroid synthesis is tightly regulated on the level of enzyme activity and gene expression of respective enzymes. A significant decrease in progesterone secretion might therefore be due to a direct inhibition of the CYP-enzyme responsible for its biosynthesis and/or accompanied by gene expression changes of relevant genes. However, no gene expression changes were observed for any substance individually and in combination at concentrations up to 40 µM for any steroid biosynthesis relevant enzyme with the exemption of *CYP19* for prochloraz and prochloraz containing mixtures (Figure 2 and Figure 3). Prochloraz increased *CYP19* mRNA 2.5-fold (*p* ≤ 0.01) individually and 2-fold (*p* ≤ 0.01) and 3.5-fold (not significant) in the combinations respectively. The observed increase in mRNA expression corresponds well with the elevated estradiol production described above.

### 3.4. Effects of Triazole Fungicides Individually and in Combination on Gene Expression of Selected Xenobiotic Metabolising Enzymes

**Figure 4:**
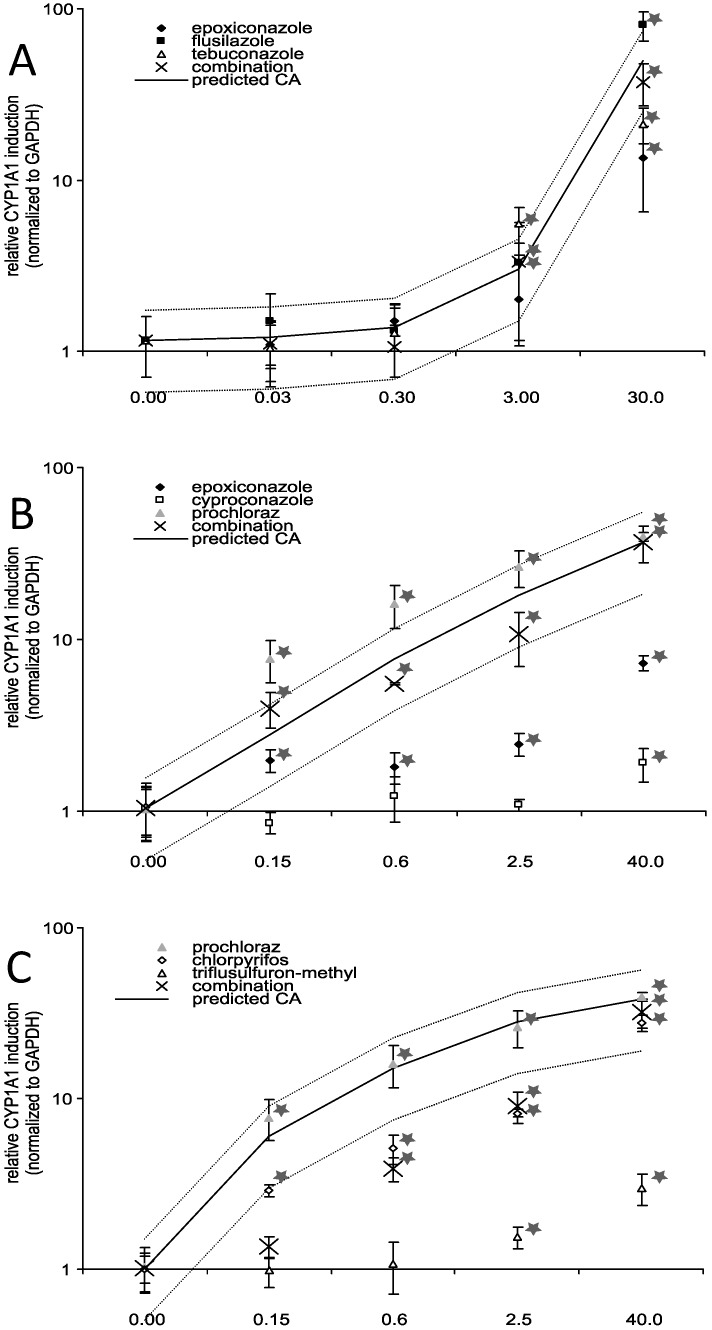
Influence of substance treatment alone or in combination on *CYP1A1* mRNA expression. Jeg-3 cells were grown in 6-well cell culture plates for 24 h. Thereafter, the cells were incubated with the substances in concentrations as indicated for 48 h individually and in combination: (**A**) flusilazole, epoxiconazole and tebuconazole, (**B**) epoxiconazole, cyproconazole and prochloraz, (**C**) prochloraz, chlorpyrifos, triflusulfuron-methyl. Finally, the RNA was isolated and reverse transcription performed as described. The data represent the mean ± SD for three independent experiments. Concentration values given for combination treatments reflect total concentrations of all three components. Predicted additivity (CA) indicates the calculated combination effect when additivity is assumed. The upper and the lower lines around the predicted CA indicate the MDR (model deviation ratio) based on the predicted CA. Concentrations are given in µM. * Indicates significance with p ≤ 0.05.

Despite the limited effects on gene expression of CYP-enzymes related to steroid biosynthesis, a clear dose response effect on the induction of *CYP1A1*, a CYP-enzyme important for xenobiotic metabolism was evident for all triazole fungicides except cyproconazole as well as for prochloraz and chlorpyrifos (Figure 4). At the concentration level 40 µM, triflusulfuron-methyl had only a very slight induction potential (3-fold, *p* ≤ 0.05). However, there was a significant difference in the potency of the triazole fungicides to increase *CYP1A1* expression. The strongest induction occurred with 40 µM prochloraz (40-fold, *p* ≤ 0.01) and chlorpyrifos (27-fold, *p* ≤ 0.01), 30 µM tebuconazole (21-fold, *p* ≤ 0.01) and 30 µM flusilazole (80-fold, *p* ≤ 0.01), while there was a moderate increase with 30 µM epoxiconazole (approx. 10-fold, *p* ≤ 0.01). Cyproconazole increased the expression slightly at the highest concentration level of 40 µM (2-fold, *p* ≤ 0.05). The combination experiment showed additive effects for the triazole fungicides. Combined exposure to a mixture of epoxiconazole, cyproconazole and prochloraz resulted in a 36-fold (*p* ≤ 0.01) induction. The combination containing prochloraz, chlorpyrifos and triflusulfuron-methyl showed combination effects and resulted in a 31-fold (*p* ≤ 0.01) induction in the highest concentration group. Nevertheless, in the lower concentration groups the combination effect was well below the MDR, indicating subadditive effects at lower concentrations. Since *CYP1A1* is commonly used as an indicator for AhR-dependent transactivation, other AhR-regulated genes were analysed. However, we observed no significant gene expression changes in commonly AhR dependent genes like *UGT2B*s and *MRP1* (data not shown). In addition, there was no detectable gene expression of the AhR dependent *CYP1A2* in Jeg-3 cells. Hence, we investigated whether the observed effect on *CYP1A1* induction caused by substances individually and in combination was mediated by AhR.

### 3.5. AhR Activation and AhR Dependent Gene Expression

**Figure 5:**
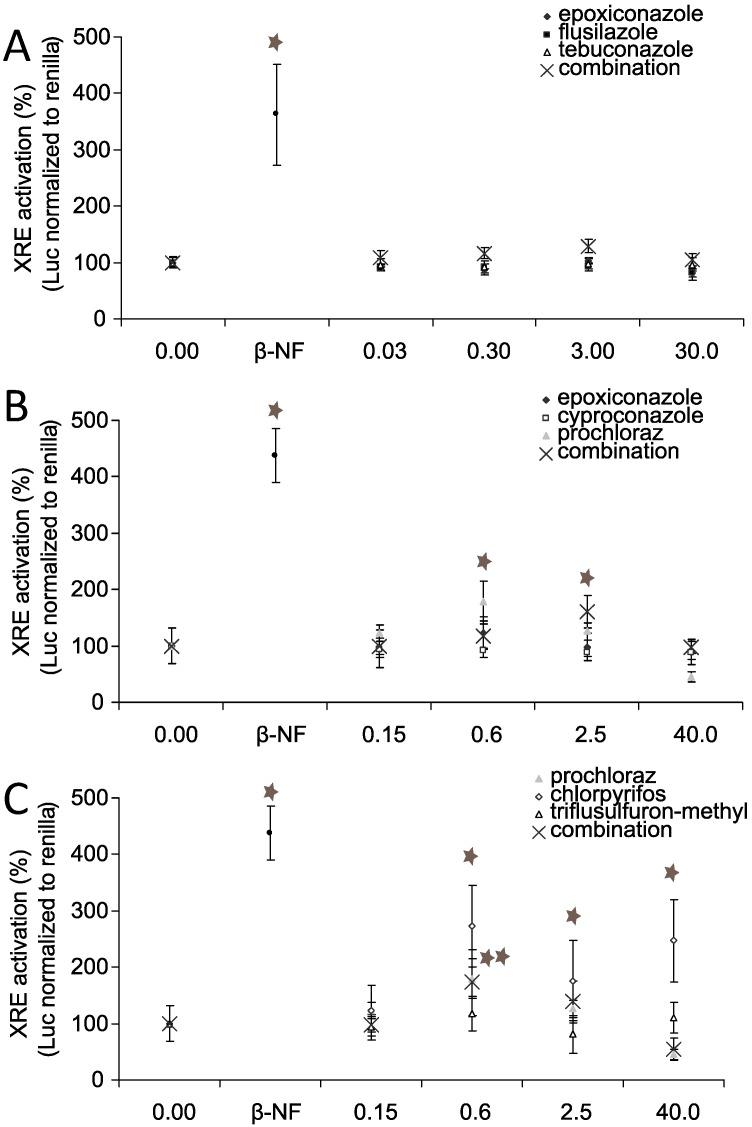
Substance effects on AhR transactivation. Jeg-3 cells were grown in 96-well cell culture plates for 24 h. Thereafter, the cells were transiently transfected with xenobiotic response element (XRE) luciferase reporter gene and renilla reporter gene (40:1) and TransIT-2020 in a ratio 1:3 for 20 h. Finally, the cells were incubated with the substances individually and in combination in concentrations as indicated: (**A**) flusilazole, epoxiconazole and tebuconazole, (**B**) epoxiconazole, cyproconazole and prochloraz, (**C**) prochloraz, chlorpyrifos, triflusulfuron-methyl. Cells were lysed and luciferase activity was measured as indicated by the manufacturer. The data represent the mean ± SD for two independent experiments (*n* = 6). Concentration values given for combination treatments reflect total concentrations for all three components. Concentrations are given in µM.

To evaluate weather the induction of *CYP1A1* was AhR dependent, we examined the ability of (tri)azoles to activate the AhR using a reporter gene assay. Figure 5 shows the results of a Dual luciferase assay conducted with Jeg-3 cells transiently transfected with an AhR reporter construct: No significant activation was observed at 0.15 µM or 0.3 µM for any of the tested substances. Prochloraz as well as chlorpyrifos activated the AhR at 0.6 µM (180% and 250% of control, resp.; *p* ≤ 0.05) as did the positive control β-naphtoflavone (450% of control). The dose response curve for prochloraz had a peak at 0.6 µM, followed by a decrease in response to 40% of control at 40 µM. The activation of AhR by chlorpyrifos remained at about 250% of control (*p* ≤ 0.05) at 40 µM, showing no change from the response at 0.6 µM.

**Figure 6:**
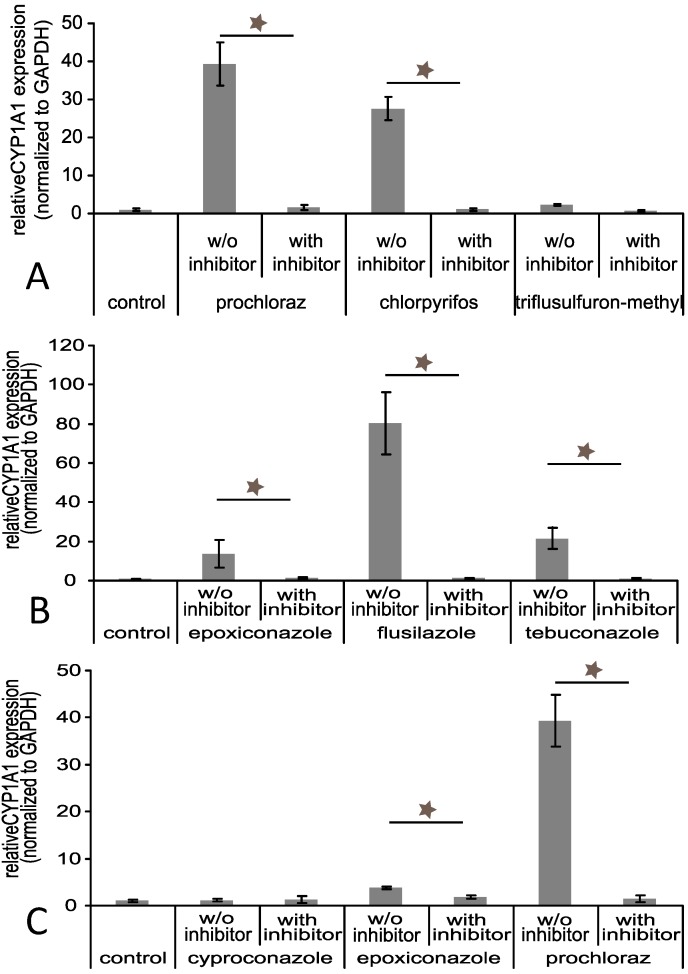
*CYP1A1* mRNA expression after substance treatment with or without AhR inhibitor. Jeg-3 cells were grown in 6-well cell culture plates for 24 h. Thereafter, the cells were incubated with 10 µM of the AhR specific inhibitor CH223191 for 4 h before incubation with the substances in total concentrations of 30 and 40 µM as indicated for 48 h individually and in combination: (**A**) 30 µM for epoxiconazole, tebuconazole and flusilazole, (**B**) 40 µM for epoxiconazole, cyproconazole and prochloraz, (**C**) 40 µM for prochloraz, chlorpyrifos and triflusulfuron-methyl. Finally, the RNA was isolated and reverse transcription performed as described. The data represent the mean ± SD for three independent experiments. * Indicates significance with p ≤ 0.05.

The combination of prochloraz, chlorpyrifos and triflusulfuron-methyl, the latter having had no activation capacity, showed cumulative patterns with a peak at 0.6 µM and 2.5 µM (160% of control at 2.5 µM; *p* ≤ 0.05) and a return to control level at 40 µM (total concentration of all three substances). While cyproconazole and epoxiconazole showed no activation properties, the combination, which contained 1/3 of prochloraz, peaked at 2.5 µM (160% of control; *p* ≤ 0.05) and showed a decrease in effect at higher concentrations. Flusilazole and tebuconazole, like epoxiconazole, were not able to activate the AhR. The same could be observed for the combination of these substances. Taken together, only prochloraz and chlorpyrifos were able to activate AhR in the reporter gene assay, but none of the other substances (triazoles) despite their ability to increase *CYP1A1* expression. However, treatment of the cells with 10 µM of the specific AhR inhibitor CH223191 prior to pesticide incubation prevented the *CYP1A1* induction for all substances, including those, that were not able to activate the AhR in the luciferase assay (Figure 6).

## 4. Discussion 

One important function of the placenta is the biosynthesis of the steroid progesterone to maintain pregnancy. Our study showed a significant concentration dependent decrease in secreted progesterone in human Jeg-3 trophoblast like cells after triazole or prochloraz treatment in high concentrations, but not after treatment with the herbicide triflusulfuron-methyl or the insecticide chlorpyrifos. Triazoles are primarily known to inhibit the CYP-enzyme aromatase (CYP19) as well as CYP17 [16,42]. Both are located downstream of progesterone in the steroid-hormone biosynthesis pathway. Hence, their inhibition would not explain the observed effect. Furthermore, the presence of active CYP17 in the human trophoblast is heavily disputed [43,44]. Another explanation would be an increased catabolism of progesterone or an inhibition of enzymes important for biosynthesis of progesterone, such as CYP11A1 that converts cholesterol to pregnenolone. An inhibition of this enzyme by triazoles has not been reported so far, but seems not unlikely considering the broad spectrum of CYP-enzymes unspecifically inhibited by triazoles.

In one study conducted with pregnant rats an increase of progesterone in maternal serum after exposure to epoxiconazole or tebuconazole was observed [16]. However, a second study on epoxiconazole in a similar strain of rats described a decrease in serum progesterone levels of dams [45]. Our results would be in line with the observations of the latter study. However, the limits of the chosen *in vitro* approach and potential species differences should be considered. The placenta varies in structure as well as in specific functions across species [46,47]. While it is the main producer of progesterone in humans in the second and third trimester, in rat progesterone is produced in the ovary over the whole gestation period [46,48,49].

Beside the progesterone production, we were also able to observe changes in the estradiol production in Jeg-3 cells after treatment with prochloraz and triflusulfuron-methyl. The estradiol production of Jeg-3 cells was found to be quite low. Nevertheless, via a sensitive ELISA we were able to quantify estradiol in the supernatant. In our study, triflusulfuron-methyl significantly decreased estradiol, which is consistent with the known properties of aromatase inhibition [39]. In contrast, the azole-fungicide prochloraz has previously been shown to reduce estradiol concentrations *in vitro* in the human adrenal cell line H295R and *in vivo* in rats via the inhibition of aromatase activity [10,11,20]. However, in our study prochloraz significantly increased the estradiol concentration in Jeg-3 cells, possibly due to the increase of *CYP19* gene expression, which was observed to correlate clearly with the estradiol increase and might serve as an explanation for the finding.

With respect to potential cumulative effects of pesticides sharing a similar mode of action it can be noted that the four triazole fungicides showed an additive effect regarding progesterone production (see also Table 2 for details). A combination of triazoles with the imidazole prochloraz also showed additive effects. Since prochloraz—like the triazoles—is a fungicide designed to inhibit the CYP-enzyme ergosterol demethylase, it can be assumed that its mode of action in terms of CYP inhibition is similar to the triazoles. Hence, additivity would be in line with the expected combination effects suggested by EFSA [14]. Accordingly, for substances with independent mode of action, no such additive effects would be expected. In fact, chlorpyrifos and triflusulfuron-methyl had no effect on progesterone production (Table 2). In combination with prochloraz there was no additive response observed. Thus, in this specific case the substances with different mode of actions acted as if given individually.

**Table 2:** Summary of effects on the endpoints progesterone synthesis and *CYP1A1* mRNA expression. Substances affecting a similar molecular endpoint revealed additive effects for this specific endpoint. DMI: (ergosterol)demethylase inhibitor; ↓ significantly (*p* < 0.05) down-regulated; ↑ significantly (*p* < 0.05) up-regulated; = no changes observed.

	Substance Class	Progesterone Synthesis	Endpoint Specific Additive Effect?	*CYP1A1* Expression	Endpoint Specific Additive Effect?
Cyproconazole	Triazole (DMI-fungicide)	↓	Yes	=	No
Epoxiconazole	Triazole (DMI-fungicide)	↓	Yes	(↑)	Yes
Flusilazole	Triazole (DMI-fungicide)	↓	Yes	↑	Yes
Tebuconazole	Triazole (DMI-fungicide)	↓	Yes	↑	Yes
Prochloraz	Imidazole (DMI-fungicide)	↓	Yes	↑	Yes
Chlorpyrifos	Organophosphate- insecticide	=	No	↑	Yes
Triflusulfuron-methyl	Triazinylsulfonyl-urea-herbicide	=	No	=	No

This was, however questionable when effects of the substances on a different endpoint (*CYP1A1* expression) were analysed. Here, also the effect of the organophosphate chlorpyrifos may have stacked with the *CYP1A1* induction caused by prochloraz at least in the highest concentration group. However, prochloraz may well have been the driving substance for the combination effect in this mixture. Hence, for this specific endpoint and concentration level, additive effects might be observed also for substances from very different pesticide classes—most likely due to their common ability to activate the AhR. Substances normally reveal their toxic properties by interaction with different receptors in different tissues at different time points and concentration levels. For example, an organophosphate, which is primarily designed to inhibit acetylcholine esterase may well also be an AhR activator and thus act additively with other substances activating the same receptor even if their pesticidal mode of action differs.

While we could find an AhR dependent induction of *CYP1A1* expression, there was no increase of other genes, which are commonly dependent on AhR activation. This is consistent with the literature regarding the trophoblast, where *CYP1A2* is commonly not detectable and UGTs as well as different drug transporters are not inducible by AhR activation [50,51] in primary human trophoblast cells as well as in Jeg-3 cells. Overall, triflusulfuron-methyl and cyproconazole exhibited the lowest capacity to increase *CYP1A1* and were not able to activate the AhR in the transactivation assay. The other substances clearly increased *CYP1A1* significantly, independently of their substance class. Nevertheless, only prochloraz and chlorpyrifos were able to activate the AhR. On the other hand, all substances that were able to increase *CYP1A1* could be inhibited by an AhR specific inhibitor, indicating that, regardless of the missing AhR activation capacity, the *CYP1A1* increase was AhR dependent. It is unclear why some of the substances failed to activate the AhR in the transactivation assay. AhR is known to play a role in the adaptive response to xenobiotics. Recent studies indicate an important role for AhR in pregnancy, as well as fetal growth and neonatal survival. Increased AhR expression upon xenobiotic stimulation leads to morphological changes in the placenta vascularisation, which is not apparent in AhR deficit mice [32]. For some substances, this has shown to have an effect on the dilatation of the maternal blood sinusoids and the maternal blood flow. AhR activation and high *CYP1A1* expression in the placenta have been associated with poor fetal growth and neonatal survival [33,50]. We showed that substances of different classes with quite different pesticidal mode of action were able to induce CYP1A1 expression and thus may contribute to a combined effect regarding this endpoint. It seems reasonable to assume that also other substances may contribute to this effect.

One should be aware that the aim of this study was to investigate mixture effects, thus, we applied rather high concentrations to reach an effect level. Often, when mixture effects are observed in *in vitro* models and then sought-out in an *in vivo* model, much higher concentrations are needed for similar effects, if an effect occurs at all. This may be due to ADME (absorption, distribution, metabolism and excretion) related issues *in vivo* as well as due to the ability for homeostatic regulation of the whole organism.

## 5. Conclusions 

In summary, the results presented in this study have several implications: firstly, combination effects by substances with dissimilar pesticidal mode of actions are clearly possible if these substances affect a common endpoint in mammalian systems. Secondly, absent combination effects, like the failing of chlorpyrifos and triflusulfuron-methyl to influence the progesterone production, might be used in concurrence with a battery of other *in vitro* assay for an adjustment e.g., of cumulative assessment groups. Thirdly, detected combination effects *in vitro*, that might be of concern for the human health, should be further investigated regarding their relevance *in vivo*.
